# Genetic Analysis in Grain Legumes [*Vigna radiata* (L.) Wilczek] for Yield Improvement and Identifying Heterotic Hybrids

**DOI:** 10.3390/plants11131774

**Published:** 2022-07-04

**Authors:** Md. Golam Azam, Umakanta Sarker, Md. Amir Hossain, Md. Shahin Iqbal, Md. Rafiqul Islam, Md. Faruk Hossain, Sezai Ercisli, Raziye Kul, Amine Assouguem, Arwa Abdulkreem AL-Huqail, Hanan R. H. Mohamed, Ilaria Peluso

**Affiliations:** 1 Pulses Research Centre, Bangladesh Agricultural Research Institute, Ishurdi, Pabna 6620, Bangladesh; azam0r@yahoo.com (M.G.A.); mdshahin.iqbal@research.uwa.edu.au (M.S.I.); 2 Department of Genetics and Plant Breeding, Faculty of Agriculture, Bangladesh Agricultural University, Mymensingh 2202, Bangladesh; amirgpb@bau.edu.bd; 3 Department of Genetics and Plant Breeding, Faculty of Agriculture, Bangabandhu Sheikh Mujibur Rahman Agricultural University, Gazipur 1706, Bangladesh; 4 School of Agriculture and Environment, University of Western Australia, Crawley, WA 6009, Australia; 5 Regional Agricultural Research Station, Bangladesh Agricultural Research Institute, Ishurdi, Pabna 6620, Bangladesh; rafiq_bari2@yahoo.com (M.R.I.); farukgolap@gmail.com (M.F.H.); 6 Department of Horticulture, Faculty of Agriculture, Ataturk University, Erzurum 25240, Turkey; sercisli@gmail.com (S.E.); raziye.kul@atauni.edu.tr (R.K.); 7 Laboratory of Applied Organic Chemistry, Faculty of Sciences and Technologies, Sidi Mohamed Ben Abdellah University, Route d’Imouzzer, Fez P.O. Box 2202, Morocco; assougam@gmail.com; 8 Department of Biology, College of Science, Princess Nourah bint Abdulrahman University, P.O. Box 84428, Riyadh 11671, Saudi Arabia; aaalhuqail@pnu.edu.sa; 9 Zoology Department, Faculty of Science, Cairo University, Giza 12613, Egypt; hananeeyra@gmail.com; 10 Research Centre for Food and Nutrition, Council for Agricultural Research and Economics (CREA-AN), 00178 Rome, Italy; i.peluso@tiscali.it

**Keywords:** GCA, heterosis, half-diallel, mungbean, SCA, yield and yield-related traits

## Abstract

Six mungbean parental lines and their fifteen F_1_s produced from half-diallel mating design were investigated for combining ability and heterosis in terms of a yield and its components. Results showed highly significant variations among the parents and F_1_s, suggesting a wide genetic variability for the studied characters. Analysis of variance indicated that genotypes mean square values, general combining ability (GCA) and specific combining ability (SCA) were highly significant (*p* ≤ 0.001) for all measured traits except for days to flowering, days to maturity, and pod length indicating genetic diversity of parents and both additive and non-additive gene effects in the inheritance of the measured traits. A higher effect of SCA than GCA for plant height and seeds per pod suggests the preponderance of non-additive gene effects in the expression of characters. Based on *per se* performance and GCA, BARI Mung-1, PS-7, and BMXK1-14004 were the best general combiners for yield per plant. In the context of SCA, hybrids BMXK1-14004 × Sonali mung, BMXK1-14004 × PS-7, BMXK1-14004 × BINA Mung-8, Sukumar × PS-7, and BARI Mung-1 × BINA Mung-8 were good specific combiners. BMXK1-14004 × Sonali mung and BMXK1-14004 × PS-7 were the best heterotic hybrids for yield and yield-contributing traits. These parents and crosses could be utilized for further use in breeding programs to improve yields in mungbean crops.

## 1. Introduction

Grain legumes, also known as mungbeans (*Vigna radiata* L. Wilczek), are a short-duration crop cultivated in major cropping systems that provides edible, nutritive, and non-bombastic food values compared with other pulses and comprises a significant wellspring of grain-based diets in Asia [1]. It is a rich source of protein with an essential amino acid profile and is wealthy in lysine [2]. Access to mungbean protein may improve the plasma lipid profile by normalizing insulin affectability [3]. It also contains unsaturated fats, which advance the development and health of humans [4]. 

The mungbean growing area in the world is about 7.3 million ha, and the mean yield is 721 kg/ha. India and Myanmar account for 30% of the worldwide output of 5.3 million tons [5]. Mungbean is the second-most important pulse crop in Bangladesh, with a total area and production of 0.24 million ha and 0.28 million tons, respectively, with a national average production of 1160 kg per ha [6]. Planting in marginal land, low yield potential, indeterminate growth habits, canopy design, low partitioning efficiency, and other biotic and abiotic stresses reduce the mungbean yield in Bangladesh. The main constraints for achieving higher yields are the lack of exploitable genetic variability, appropriate ideotype for various crop systems, unavailability of improved seed quality, and a narrow genetic base as a result of repeated use by a small group of parents with a strong link in crossing programs [7]. Plant breeders create variability to select superior genotypes in crop improvement programs [8,9,10]. The achievement of any breeding program depends on the degree of diversity and variability. A wide range of variability and diversity across the mungbean germplasm was reported in the literature [11,12,13]. The extent of variation in the heritable components is crucial for growers in crop improvement programs [14,15]. In the literature, several papers detailing the qualitative and quantitative traits in terms of diversity and variability were reported, such as agronomic traits [16], minerals [17], grain yield [18], pigments [19], proximate compositions [20], vitamins [21], flavonoid content [22] phenolics [23], and antioxidant activity [24,25,26,27,28]. Hence, there is an urgent need to boost production and productivity for food and nutritional security, which improves the genetic yield potential of current varieties by restructuring their plant type. As a result, plant breeders must employ heterosis to create superior hybrids for their plants [29].

A varietal improvement program depends on the selection of genotypes and their high combining ability. Combining ability is an influential tool for identifying the best combiner, application of suitable crosses to assemble required genes, or accomplishing heterotic segregates [30,31,32]. The research of combining ability in diallel patterns is beneficial since it elucidates the nature and magnitude of different types of gene activities [33]. The majority of diallel investigations on gene activity and combining ability in mungbeans have shown a high prevalence of variability due to their general combining ability (GCA) [34]. The impact of the GCA is limited by additive genetic interactions, while the SCA effect is constrained by non-additive genetic interactions [35].

Heterosis provides the breeder instructions for selecting the optimum cross-combination in the first generation. In addition, the extent of heterosis offers the foundation for genetic information and guidance for selecting desirable hybridization parents. Several professionals have used the combining ability to analyze the genetic impact and genetic value of parents in various crops. The findings were well matched with earlier publications [11,12,13,34]. Combining ability and heterosis has been successfully used to reproduce mungbeans [11,12,13]. However, study data on mungbean are few in number. Considering these issues, this trial was undertaken to determine the degree of combining ability and heterosis of mungbean lines for yield, as well as the nature of gene activity associated with mungbean genotypes using a diallel mating design.

## 2. Results and Discussions

### 2.1. Analysis of Variance (ANOVA) for Combining Ability

Highly significant ANOVA for all the parameters was observed, which indicated the preponderance of genetic variations across the genotypes and justified the inclusion of the genotypes for a combining ability study. A wide range of variability was also reported in different mungbean genotypes [11,12,13,29], rice germplasm [36,37,38], maize [39], and other crops [40,41]. The analysis of variance (Table 1) shows highly significant variations among the parents and offspring for all the studied characters indicating the presence of genetic variability in the material under study. The analysis of variance for combining ability and asses of genetic difference components of each character is presented in Table 1. The statistical analyses discovered exceptionally high differences between the parents and their hybrids (F_1_) for all the characters (Table 1). These findings provide proof of the closeness of a highly significant amount of genetic variability among the mungbean parents and their respective hybrids (F_1_), which may encourage genetic improvement utilizing such genetic pools of mungbeans. These outcomes were in agreement with those reported by Latha et al. [11], Kumar et al. [42], Sopan et al. [13], and Viraj et al. [34].

The mean square of general combining ability (GCA) and specific combining ability (SCA) were significant for all the characters. SCA, DF, DM, and pod length had no significant difference (Table 1) and showed non-additive gene effects for the expression of these characters. The GCA fluctuation contains an additive epistasis effect, while the SCA difference contains a non-additive effect as outlined by Griffing [43]. Thus, the significant assessments of both GCA and SCA variances indicated that both the additives and non-additive nature of gene actions were engaged in controlling these characters in studied mungbean genotypes. These outcomes affirmed those discoveries by Reddy et al. [33] Nath et al. [44], Viraj et al. [34], and Sopan et al. [13]. The assessments of differences may be because of higher general combining ability than the specific combining ability for all the characteristics except plant height and seeds per pod brought up to be the dominance of non-additive gene effects in the outflow of these characters.

The GCA/SCA proportion was utilized to explain the idea of the genetic differences involved. The GCA/SCA proportions were greater than the unity for the number of pods per plant, pod length, and seed yield per plant, demonstrating that the additive types of the gene actions were increasingly significant in the inheritance of these attributes than non-additive types. However, the GCA/SCA proportions were lower than unity for plant height and number of seeds per pod, demonstrating the prevalence of non-additive gene effects in the expression of these characteristics, which were corroborative with findings concerning rice [45,46,47]. Consequently, the selection can be fruitful in the improvement of our mungbean materials. In any case, it could be emphasized that the GCA/SCA proportion may not generally change the appearance of gene action for specific characters. The current outcomes are corroborative of the previous findings in mungbeans [11,12,48].

### 2.2. Mean Evaluation of Parents and Their Crosses

The mean of yield and yield-contributing characters for parents and their F_1_s are presented in Table 2. In the case of days to flowering, the P_2_ × P_6_ cross indicated early flowering and P_3_ × P_6_ as late flowering. The cross P_4_ × P_5_ was selected as early maturing with 66.5 days, and the P_5_ × P_6_ cross was late maturing with 79.5 days for maturity. The parent P_4_ (Sukumar) had the tallest plants (61.2 cm), while the crosses P_3_ × P_6_ and P_3_ × P_5_ recorded the lowest values 33.6 and 34.5 cm, respectively. The number of pods per plant ranged from 12 to 42, whereas F_1_ of P_1_ × P_4_ was the most superior and F_1_ of P_5_ × P_6_ produced the lowest number of pods per plant. P_1_ × P_3_ produced the minimum number of seeds per pod (45), followed by P_2_ (BARI Mung-1), and a maximum (64) was recorded in crosses P_3_ × P_4_. The P_4_ × P_5_ had the maximum pod length (8.35 cm), while Sonali mung (P_6_) scored the lowest (5.60 cm). The higher seed yield per plant (9.9 g) was found from (P_1_ × P_5_), which was a predominant cross-combination and lower yield from the P_6_ parent (6 g). These results could affirm the chance of determination for these characters through the hybridization of particular parents. In addition, it suggests plant breeders assemble future breeding work for high yield in mungbean crops.

### 2.3. General Combining Ability Effects

The GCA reveals the additive nature of gene action. In the present investigation of mungbeans, the highly significant and positive extent of GCA for the number of pods per plant, pod length, number of seeds pods^−1,^ and seed yield per plant desired, while profoundly significant and negative values for days to maturity and plant height are suitable (Table 3). According to Mondal et al. [49], the synchrony of pod maturity in mungbeans is achievable when the plant is short in height and the same type or has one branch; thus, emphasis should be placed on the development of this attribute in mungbean-breeding programs. Parent P_1_, P_5,_ P_4_, and P_2_ showed a highly significant and positive effect of GCA on the number of pods per plant, plant height, and pod length seed yield per plant, respectively, indicating that those genotypes could be used as a good general combiner in the breeding program. In addition, P_2_ and P_5_ also were shown to be a good parental combiner for dwarfness in mungbeans, having profoundly significant and negative GCA values. As a result, it was seen that none of the parents were demonstrated to be a great general combiner for all the characteristics, while parents P_4_, P_5_, and P_2_ may be revealed as practically good general combiners. However, parent P_6_ was the very lowest general combiner for all the characters. These results are similar to previous findings of Gupta et al. [50], Patil et al. [12], Kumar and Prakash [51], and Sujatha and Kajjidoni [52]. These parents would be intensely utilized for higher yield.

### 2.4. Specific Combining Ability Effect

The SCA indicates the role of non-additive gene action in the expression of characters. A study of SCA exposed that none of the hybrids displayed a favorable SCA effect for all the studied characters (Table 4). Among fifteen crosses, P_1_ × P_6_, P_3_ × P_6,_ and P_1_ × P_3_ gave a highly significant and negative assessment of SCA effects for days to maturity and plant height (Table 4). For the number of pods per plant, P_1_ × P_5_, P_2_ × P_3_, P_3_ × P_6_, P_1_ × P_4,_ and P_2_ × P_4_ gave highly significant and positive SCA effects. The cross P_2_ × P_4_ gave the highly significant and positive measures of SCA effects for pod length; whereas P_2_ × P_3_, P_3_ × P_6_, P_1_ × P_5_, P_2_ × P_4,_ and P_2_ × P_5_ for the number of seeds per pod. Five crosses (P_1_ × P_6_, P_1_ × P_5_, P_4_ × P_5_, P_1_ × P_3,_ and P_2_ × P_3_) out of fifteen crosses had the significantly best SCA effect for seed yield per plant. These crosses could be utilized in breeding programs to improve studied traits. It was noteworthy that practically all the best crosses in the event of respective attributes also displayed desirable *per se* performance for individual traits. It was notable that none of the best hybrids had included the two parents with good × good GCA effects showing non-additive × additive interaction. Among all the crosses, just five crosses P_1_ × P_6_, P_1_ × P_5_, P_2_ × P_3_, P_3_ × P_6_, and P_2_ × P_4_ displayed the desirable extent of SCA effects for the highest five attributes out of considered seven characteristics, including average × poor, good × average combiners. These results are getting support from the findings of mungbean [11,12,13,37,49]. This may be because of epistasis like additive × dominance type of interaction. These crosses could be exploited to get desirable recombinants from the distinct population.

### 2.5. Heterosis

All traits showed variations between parents and cross-combinations because of heterosis, which were corroborative to the findings of mungbean [29,33]. Considerable positive heterosis compared with better-parent estimates would be enthusiasm for pods per plant, pod length, seeds per pod, and yield per plant, whereas it is helpful to have significant negative heterosis compared with better-parent esteems for days to flowering, days to maturity, and plant height. Among the fifteen combinations, eleven hybrids demonstrated negative heterosis compared with the better-parent for plant height and six for considerable days to flowering, whereas six combinations for pods per plant, eight combinations for pod length, five combinations for seeds per pod and four combinations for yield per plant had positive heterosis compared with better-parent esteems, as expected with a predominance of additive effects. The heterosis esteems assessed for explored traits in F_1_ combinations are given in Table 5.

The scale of relative heterosis was observed as −9.29% (P_2_ × P_6_) to 13.60% (P_1_ × P_5_) for days to flowering. The heterobeltiosis among the hybrids varied between −14.43% (P_2_ × P_6_) and 11.49% (P_1_ × P_5_). The information revealed that out of 15 hybrids, 9 and 5 hybrids indicated significant desirable heterosis over mid-parent and better-parent individually. The relative heterosis extended from −1.37% (P_1_ × P_6_) to 12.86% (P_1_ × P_3_) for days to maturity. Heterobeltiosis differed between −10.19% (P_1_ × P_6_) and 12.06% (P_1_ × P_3_). The ata indicated that four and six hybrids displayed significant negative heterosis over mid-parent and better-parent, respectively. All the studied characters demonstrated positive and negative mid-parent and better-parent heterosis in all the hybrids. Heterosis esteems among all the crosses for plant height differed from −31.00% to 20.27% and −43.63 to 14.69% for MP and BP heterosis, individually. Desirable highly negative MP heterosis and BP heterosis for plant height were found from the crosses P_3_ × P_5_. This heterotic effect for plant height demonstrates that short plants can be developed by utilizing this hybrid. MP and BP heterosis esteem fluctuated from −38.78% to 54.76% and −50.41 to 34.48%, respectively for pods per plant. Eleven hybrids showed positive relative heterosis and six hybrids demonstrated positive heterobeltosis for pods per plant, showing that the genes with negative effects were dominant for this attribute in the crosses. The cross P_2_ × P_3_ showed desirable positive MP (54.76%) and BP (34.48%) heterosis followed by P_1_ × P_4_ for pods per plant.

MP and BP heterosis esteem ranged from −4.23% to 36.07% and −16.05% to 38.07% for pod length, respectively. Thirteen hybrids expressed positive heterotic effects over mid-parent and eight over better-parent for pod length. The degree of heterosis for this character was similarly low, and the parental value for this characteristic was also low, which created restrictions on improving this attribute in the material utilized in this experiment. The F_1_ hybrid P_5_ × P_6_ showed the highest MP and BP heterotic effects of 36.07% and 38.07%, respectively, followed by the cross P_4_ × P_5_. Kumar et al. [7] and Srivastava and Singh [29] found similar results. In the case of the number of seeds per pod, the MP and BP fluctuated from −19.64% to 14.81% and −25.00% to 11.54%, respectively. For the number of seeds per pod, seven hybrids exceeded the mid-parent and five over the better-parent. The highest relative heterotic effect and heterobeltosis were exposed by the hybrid P_1_ × P_5_, demonstrating that this cross may be misused for improving this attribute. Similar results for seeds per pod have been found by Dhuppe et al. [53], Zubair et al. [54], Kumar et al. [7], and Yadav et al. [55].

The values of relative heterosis for the yield of hybrids ranged from −18.08% to 26.09%, and heterobeltosis extended from −31.01% to 20.20% for seed yield per plant. Among all the crosses, four hybrids exceeded both mid-parent and a better-parent. The hybrids P_1_ × P_6_ showed the highest MP, and P_1_ × P_5_ hybrids indicated the greatest BP heterotic effect, followed by the cross P_1_ × P_4_. These discoveries were similar to earlier reports by Dhuppe et al. [53], Zubair et al. [54], and Kumar et al. [7].

The current experiment demonstrates that the undesirable negative mean heterosis is observed in all the attributes for both mid-parent and better-parent heterosis except for pod length and the number of seeds per pod for mid-parent; however, the expected positive mean heterosis was reached in terms of plant height. Among these lines, a more diversified germplasm is required to be imported for use in the breeding system to improve these yield contributing characteristics in mungbean. Subsequently, the cross P_4_ × P_5_ shows high positive heterotic effects for pod length and grain yield per plant, and high negative heterotic effects for plant height may be exploited for the above characteristics to grow high-yielding mungbean cultivars.

## 3. Materials and Methods

### 3.1. Experimental Site and Climate

**Figure 1:**
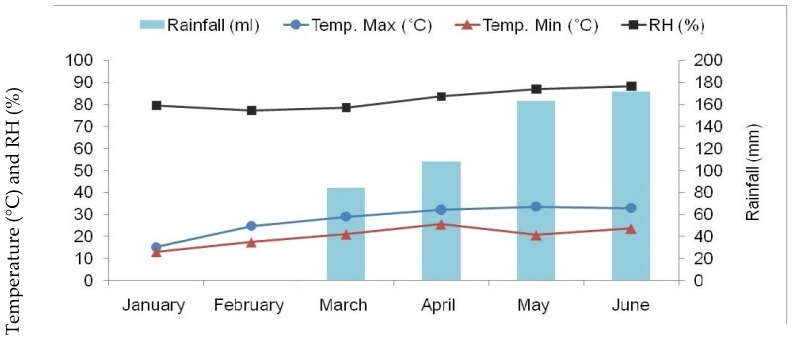
Monthly average maximum and minimum temperature (°C), relative humidity [RH (%)], and total rainfall prevailed during 2015. Temp.—temperature, Max—maximum, Min—minimum.

**Figure 2:**
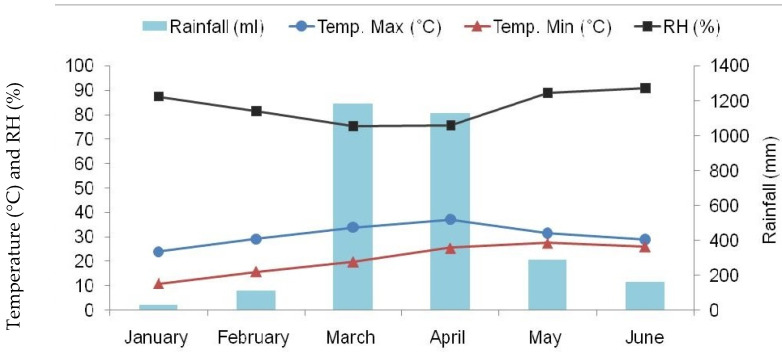
Monthly average maximum and minimum temperature (°C), relative humidity [RH (%)], and total rainfall prevailed during 2016. Temp.—temperature, Max—maximum, Min—minimum. (Source: Bangladesh Sugar Crop Research Institute).

The experiment was conducted at the pulses breeding section at Pulses Research Center (PRC) of Bangladesh Agricultural Research Institute (BARI), Ishurdi, Pabna, situated at 24.07° north latitude and 89.03° east longitude having an altitude of 11.58 m above the mean sea level. The experimental site is a part of the High Ganges River Floodplain agro-ecological zone (AEZ-11) of Bangladesh, consisting of calcareous soil. The field is clay loam with low-to-medium fertility (Table 6), and the weather data of the developing seasons are presented in Figure 1 and Figure 2.

### 3.2. Experimental Materials and Crossing Technique

The plant materials comprised six genotypes of grain legume (*Vigna radiata* L.): four locally collected lines (viz. BMXK1 14004, BARI Mung-1, BINA Mung-8 and Sonali Mung), and two exotic lines (viz. Sukumar and PS-7). The mungbean parents were selected for the crossing program based on diverge morphology, seed size, color, and tolerant ability. (Table 7). The cross-achievement rate was lower in the open field under regular natural conditions; consequently, the crosses endeavored at good field conditions for acceptable emasculation, crossing, and normal pod development in a greenhouse during spring 2015 in a half-diallel fashion (excluding reciprocals) to obtain all of the possible combinations (complete of 15 F_1_s crosses). Hand emasculation and hand pollination were used to produce the seeds of 15 hybrids (Table 8). The 15 F_1_ crosses alongside their 6 parents established 21 lines were developed in a randomized block design with three replications during the developing periods of 2016 at the PRC research field, Bangladesh Agricultural Research Institute (BARI), Ishurdi, Pabna.

### 3.3. Crop Management

Each genotype was planted by dibbling the seeds in two rows of 3 m in length, with a spacing of 30 cm between the lines and 7 cm between the plants. The land was fertilized with 20-40-20-10 N-P-K-S kg per ha as urea, triple superphosphate, muriate of potash, and gypsum, respectively, at final land preparation. After seed-sowing, flood irrigation was given to ensure seed germination. Mulching was done, and the soil outside the layers was broken. Thinning was done to maintain a single seedling per hill 20 days after sowing. Irrigation, weeding, and plant protection measures were taken as requirements during the development period, according to BARI [56] recommendation.

### 3.4. Data Collection

The data for days to flowering (DF), days to maturity (DM), plant height, number of pods per plant, pod length, number of seeds per pod, and seed yield per plant were recorded from ten randomly selected plants and then averaged to a per-plant basis. Pod length (cm) and seeds per pod were recorded on five pods selected randomly from ten plants within each genotype. The seed weight per plant was recorded in grams by weighing all seeds from the five plants and dividing them by five.

### 3.5. Statistical Analysis

The evaluations of difference for both the general and specific combining abilities and their belongings were processed by Model I (fixed-effect model) and Method II (parents and crosses, excluding reciprocals) as provided by Griffing [35]. For the combining ability, analysis of variance was performed for characteristics that demonstrated significant differences among crosses [57] (Plant Breeding Tools, 2014, International Rice Research Institute, Los Baños, Laguna) software version 1.2 utilizing R packages. The significance of the GCA effects was recorded utilizing the following equation [58,59]:

$$t_{cal}=\frac{\mathrm{GCA}\;}{{\mathrm{SE}}_{\mathrm{gca}}},\mathrm{Where},\;{\mathrm{SE}}_{\mathrm{gca}}=\sqrt{\frac{M_{e}}{rts}}$$

$$t_{cal}=\frac{\mathrm{SCA}\;}{{\mathrm{SE}}_{\mathrm{sca}}},\mathrm{Where},\;{\mathrm{SE}}_{\mathrm{sca}}=\sqrt{\frac{M_{e}}{rs}}$$

where, *M_e_* is the error mean sum of squares; *r*, *t*, and *s* are numbers of replications, parental lines, and sites, respectively; SE is the standard error.

Heterosis is expressed as a percentage increase or reduction in the F_1_ hybrid over mid-parent (average or relative heterosis) and better-parent (heterobeltiosis). Mid-parent heterosis, heterobeltiosis, and their significant tests were accomplished for each character by following the equation depicted by Abrham et al. [60].

MP Mid-parent heterosis (%) = [(F_1_ − MP)/MP] × 100

BP Better-parent heterosis (%) = [(F_1_ − BP)/BP] × 100

where F_1_ = mean of F_1_hybrid for a trait

MP = mean of mid-parents [(P1 + P2)/2] for a trait

BP = Value of better-parents for a trait

SOM = Soil organic matter

## 4. Conclusions

The experiment has been aimed at distinguishing superior parents as the best combiner and best predominant crosses as particular combiners for various characters based on different parameters, viz. *per se* performance, GCA effects, SCA effects, and prevalence of F_1_ over the mid- and better-parent. Based on combining ability analysis, the most promising parents P_2_ (BARI Mung-1) and P_5_ (PS-7) for yield per plant, pod length, and plant height; P_4_ (Sukumar) for seeds per pod and other desirable traits such as pods per plant and yield per plant for P_1_ (BMXK1-14004). The crosses *viz.*, P_1_ × P_6_, P_1_ × P_5_, P_1_ × P_3_, P_4_ × P_5,_ and P_2_ × P_3_ had distinguished as best specific cross-combinations for the majority of the yield attributes together with a few interesting traits. The crosses P_1_ × P_5_ (BMXK1-14004 × PS-7), P_1_ × P_6_ (BMXK1-14004 × Sonali mung), P_1_ × P_4_ (BMXK1-14004 × Sukumar), P_1_ × P_3_ (BMXK1-14004 × BINA Mung-8) displayed significant better-parent heterosis for seed yield per plant including its components. Consequently, these crosses could be used in further breeding programs to isolate desirable segments in terms of the mating approach followed by the selection in their segregating generations.

## Figures and Tables

**Table 1:** Analysis of variance for combining ability of different plant characters in mungbeans.

Source of Variance	d.f.	Mean Square						
		DF	DM	Plant Height	Pods per Plant	Pod Length	Seeds per Pod	Yield per Plant
Replications	2	3.45	5.54	0.25	2.49	0.03	2.78	0.03
Genotypes	20	51.78 **	44.23 **	248.19 **	265.22 **	2.74 **	256.73 **	2.66 **
Parents	5	29.12 **	66.45 **	301.60 **	73.81 **	5.65 **	80.89 **	5.29 **
Crosses	14	65.67 **	38.43 **	102.88 **	346.93 **	1.89 **	248.05 **	1.82 **
Error	40	24.54 **	24.23 **	1.58	3.39	0.51	5.58	0.22
GCA	5	14.00 **	14.43 **	72.28 **	109.02 **	1.41 *	40.06 **	1.45 *
SCA	15	5.67	10.22	85.22 **	81.26 **	0.57	100.42 **	0.52 **
Error	40	0.87	0.76	0.50	1.11	0.15	1.65	0.07
GCA/SCA		2.47	1.41	0.85	1.34	2.47	0.40	2.79

d.f.—Degrees of freedom, DF—days to flowering, DM—days to maturity, GCA—General combining ability, SCA—specific combining ability, *,** Significant at 5% and 1% level of probability, respectively.

**Table 2:** Mean ± standard deviation (sd) of days to flowering, days to maturity, plant height, pods per plant, pod length, seeds per pod, and yield per plant of parents and their F_1_ progenies.

Parents/Crosses	Days to Flowering	Days to Maturity	Plant Height	Pods per Plant	Pod Length	Seeds per Pod	Yield per Plant
**Parents**							
P_1_	43.5 ± 1.20	70.5 ± 1.17	40.5 ± 2.20	34.2 ± 2.17	8.1 ± 0.56	11 ± 1.16	7.8 ± 0.46
P_2_	43.0 ± 1.72	71.5 ± 1.46	46.4 ± 0.72	21.4 ± 0.46	8.5 ± 0.51	10 ± 1.53	8.9 ± 0.36
P_3_	42.5 ± 2.19	69.5 ± 2.08	50.6 ± 0.19	29.0 ± 1.08	8.1 ± 0.85	12 ± 2.00	7.8 ± 0.48
P_4_	43.5 ± 1.71	68.5 ± 1.88	61.2 ± 0.71	33.4 ± 0.88	6.2 ± 0.24	12 ± 3.00	7.1 ± 0.83
P_5_	42.0 ± 2.51	71.5 ± 1.53	38.8 ± 1.51	15.0 ± 0.53	9.9 ± 0.70	11 ± 2.00	9.9 ± 0.55
P_6_	48.5 ± 2.00	78.5 ± 1.70	35.2 ± 1.00	24.2 ± 0.70	6.1 ± 0.85	11 ± 1.53	6.0 ± 0.44
**Crosses**							
P_1_ ×P_2_	44.5 ± 2.40	79.5 ± 2.10	41.66 ± 1.40	34.20 ± 1.10	8.00 ± 1.12	10 ± 2.08	8.60 ± 0.25
P_1_ ×P_3_	44.0 ± 2.38	79.0 ± 1.45	37.00 ± 1.38	26.75 ± 0.45	8.06 ± 0.45	9 ± 2.00	8.25 ± 0.40
P_1_ × P_4_	44.5 ± 2.92	76.5 ± 1.85	46.75 ± 0.92	42.75 ± 0.85	7.38 ± 0.54	10 ± 2.03	7.80 ± 0.30
P_1_ × P_5_	48.5 ± 2.47	68.5 ± 1.78	43.25 ± 1.47	36.25 ± 0.78	8.88 ± 0.72	12 ± 2.00	9.90 ± 0.16
P_1_ × P_6_	49.0 ± 2.31	70.5 ± 2.00	39.50 ± 0.31	36.25 ± 1.00	8.60 ± 0.49	12 ± 2.52	8.70 ± 0.55
P_2_ × P_3_	44.5 ± 3.05	67.5 ± 3.08	37.00 ± 2.05	39.00 ± 2.08	9.30 ± 0.71	12 ± 3.00	8.90 ± 0.43
P_2_ × P_4_	43.5 ± 1.76	76.5 ± 2.55	46.75 ± 0.76	31.60 ± 1.55	8.30 ± 0.68	12 ± 1.53	6.90 ± 0.10
P_2_ × P_5_	43.5 ± 1.62	76.5 ± 2.20	43.25 ± 0.62	18.75 ± 1.20	9.25 ± 0.78	10 ± 2.08	8.60 ± 0.47
P_2_ × P_6_	41.5 ± 1.79	76.0 ± 1.80	39.50 ± 0.79	26.25 ± 0.80	8.60 ± 0.65	10 ± 1.00	6.75 ± 0.23
P_3_ × P_4_	43.5 ± 2.27	76.5 ± 3.67	49.75 ± 2.27	31.75 ± 2.67	8.30 ± 1.01	13 ± 2.52	6.75 ± 0.45
P_3_ × P_5_	42.5 ± 2.11	76.5 ± 2.34	34.50 ± 1.11	25.00 ± 1.34	8.30 ± 0.72	11 ± 2.52	7.25 ± 0.81
P_3_ × P_6_	49.0 ± 2.22	78.5 ± 1.62	33.60 ± 1.22	35.20 ± 0.62	6.80 ± 0.56	11 ± 1.00	6.30 ± 0.46
P_4_ × P_5_	47.5 ± 2.15	66.5 ± 2.51	57.50 ± 1.15	25.50 ± 1.51	10.40 ± 0.81	12 ± 1.53	9.20 ± 0.47
P_4_ × P_6_	43.0 ± 1.77	74.5 ± 1.79	44.50 ± 0.77	25.75 ± 0.79	6.70 ± 0.42	11 ± 2.08	6.50 ± 0.34
P_5_ × P_6_	44.0 ± 1.27	79.5 ± 1.75	43.33 ± 0.27	12.00 ± 0.75	8.30 ± 0.51	12 ± 3.21	6.83 ± 0.27
Mean	44.57	73.93	43.36	28.77	8.19	12.90	7.84
LSD at 5%	3.05	2.67	2.07	3.04	1.16	1.90	0.77

**Table 3:** Estimates of general combining ability effects of the parents for yield and different characters.

Parents	Characters						
	DF	DM	Plant Height	Pods per Plant	Pod Length	Seeds per Pod	Yield per Plant
P_1_	−0.37	−0.03	2.86 **	6.11 **	−0.23	−0.24	0.34 *
P_2_	−1.28 **	1.54 **	−2.92 **	−1.49 **	0.35 *	0.51	0.36 *
P_3_	−0.55	−0.86	−2.25 **	1.68 **	0.09	0.67	−0.05
P_4_	2.54 **	2.24 **	3.97 **	0.75	−0.50 *	3.68 **	−0.50 **
P_5_	−0.27	1.22 *	−2.61 **	−3.21 **	0.59 **	−2.21 **	0.46 **
P_6_	−0.26	−0.81	0.93 **	−3.86 **	−0.29	−2.43 **	−0.51 **
SE Gi	0.27	0.51	0.23	0.34	0.13	0.41	0.08
SE Gi-Gj	0.42	0.69	0.35	0.53	0.20	0.64	0.13

DF—days to flowering, DM—days to maturity, *, ** Significant at 0.05 and 0.01 levels of probability, respectively.

**Table 4:** Specific combining ability effects of the different 15 crosses for yield and its related traits in mungbean.

Crosses	Characters						
	DF	DM	Plant Height	Pods per Plant	Pod Length	Seeds Per Pod	Yield per Plant
P_1_ × P_2_	−0.75	0.85	2.74 **	0.45	0.04	−16.45 **	−0.01
P_1_ × P_3_	2.83 **	2.01	−2.57 **	−10.64 **	0.54	−2.95 **	0.77 *
P_1_ × P_4_	−0.09	−0.83	0.95	6.37 **	0.72	0.05	0.42
P_1_ × P_5_	0.26	−0.39	4.03 **	24.31 **	−1.42	5.93 **	0.82 *
P_1_ × P_6_	2.22 **	−3.08 *	−3.27 **	−6.10 **	−1.11 **	2.83	0.84 *
P_2_ × P_3_	1.38	3.35 *	7.58 **	9.35 **	−1.14 *	6.30 **	0.68 *
P_2_ × P_4_	2.25 **	6.68 ***	0.38	2.65 *	0.53 *	4.63 **	−0.93 **
P_2_ × P_5_	0.08	5.23 **	9.74 *	−6.53 **	0.28	3.18 *	−0.05
P_2_ × P_6_	2.68 **	2.04	1.59	2.28	0.41	2.74	−0.74 *
P_3_ × P_4_	3.02 **	−0.08	8.08 **	−0.20	0.83	−11.20 **	−0.37
P_3_ × P_5_	−0.64	−0.56	0.23	−5.64 **	−0.26	−20.66 **	−0.85 *
P_3_ × P_6_	0.44	−0.86	−3.23 **	8.02 **	0.01	6.24 **	−0.71 *
P_4_ × P_5_	4.36 ***	2.48	17.16 **	−3.24 *	−0.26	−2.33	0.78 **
P_4_ × P_6_	4.36 ***	5.29 ***	0.66	−0.76	−0.37	−4.76 **	0.19
P_5_ × P_6_	−0.34	−0.71	5.85 **	−10.06 **	0.56	−11.22 **	−0.45
SE S_ij_	0.74	1.29	0.63	0.93	0.35	1.14	0.23
SE S_ij_-S_ik_	1.11	1.93	0.94	1.39	0.52	1.70	0.35
SE S_ij_-S_kl_	1.03	1.79	0.87	1.29	0.48	1.57	0.32

DF—days to flowering, DM—days to maturity, *, **, *** Significant at 0.05, 0.01, and 0.001 levels of probability, respectively.

**Table 5:** Heterosis values (%) over mid-parent and better-parent for important characters of fifteen mungbean crosses.

**Crosses**	**DF**		**DM**		**Plant Height**		**Pods per Plant**	
	**MP (%)**	**BP (%)**	**MP (%)**	**BP (%)**	**MP (%)**	**BP (%)**	**MP (%)**	**BP (%)**
P_1_ × P_2_	2.89 **	2.30 *	11.97 **	11.19 **	−4.12 **	−10.91 **	23.02 **	−2.92 **
P_1_ × P_3_	2.33 *	1.15 NS	12.86 **	12.06 **	−18.77 **	−29.86 **	−15.35 **	−21.78 **
P_1_ × P_4_	2.30 *	2.30 *	10.07 **	8.51 **	−8.06 **	−28.42 **	26.48 **	25.00 **
P_1_ × P_5_	13.45 **	11.49 **	−3.52 **	−4.20 **	9.08 **	6.94 **	47.36 **	5.99 **
P_1_ × P_6_	6.52 **	1.03 NS	−5.37 **	−10.19 **	4.36 **	−2.64 *	24.14 **	5.99 **
P_2_ × P_3_	4.09 **	3.49 **	−4.26 **	−5.59 **	−23.71 **	−28.04 **	54.76 **	34.48 **
P_2_ × P_4_	0.58NS	2.30 *	9.29 **	6.99 **	−16.37 **	−25.85 **	15.33 **	−5.39 **
P_2_ × P_5_	2.35 *	1.16 NS	6.99 **	6.99 **	−13.50 **	−35.90 **	3.02 **	−12.38 **
P_2_ × P_6_	−9.29 **	−14.43 **	1.33 NS	−3.18 **	6.76 **	1.89 NS	15.13 **	8.47 **
P_3_ × P_4_	1.16 NS	−2.30 *	10.87 **	10.07 **	−11.00 **	−20.48 **	1.76 NS	−4.94 **
P_3_ × P_5_	0.59 NS	−2.35 *	8.51 **	6.99 **	−31.00 **	−8.60 **	13.64 **	−13.79 **
P_3_ × P_6_	7.69 **	1.03 NS	6.08 **	−1.27 NS	−9.19 **	−74.59 **	32.33 **	21.38 **
P_4_ × P_5_	11.11 **	9.20 **	−5.00 **	−6.99 **	15.00 **	37.40 **	5.37 **	−23.65 **
P_4_ × P_6_	−6.52 **	−11.34 **	1.36 NS	−5.10 **	20.27 **	15.41 **	−10.59 **	−22.90 **
P_5_ × P_6_	−2.76 **	−9.28 **	6.00 **	1.27 NS	17.11 **	117.11 **	−38.78 **	−50.41 **
**Crosses**	**Pod Length**		**Seeds per Pod**		**Yield per Plant**			
	**MP (%)**	**BP (%)**	**MP (%)**	**BP (%)**	**MP (%)**	**BP (%)**		
P_1_ × P_2_	2.56 *	−5.88 *	2.00 NS	−1.92 NS	2.99 **	−3.37 **		
P_1_ × P_3_	6.05 **	−0.49 NS	−19.64 **	−25.00 **	5.77 **	5.77 **		
P_1_ × P_4_	10.98 **	3.94 **	−14.04 **	−20.97 **	4.70 **	12.82 **		
P_1_ × P_5_	11.00 **	−0.22 NS	14.81 **	10.71 **	11.86 **	20.20 **		
P_1_ × P_6_	13.16 **	6.17 **	11.54 **	11.54 **	26.09 **	11.54 **		
P_2_ × P_3_	12.05 **	9.41 **	9.26 **	−1.67 NS	6.59 **	−3.37 **		
P_2_ × P_4_	12.93 **	−2.35 *	7.27 **	−4.84 **	−13.75 **	−22.47 **		
P_2_ × P_5_	6.32 **	3.93 **	−7.69 **	−14.29 **	−8.51 **	−13.13 **		
P_2_ × P_6_	17.81 **	1.18 NS	−4.00 **	−7.69 **	−9.40 **	−24.16 **		
P_3_ × P_4_	16.08 **	2.47 *	4.92 **	3.23 **	−9.40 **	−13.46 **		
P_3_ × P_5_	−2.35 *	−6.74 **	−6.90 **	−10.00 **	−18.08 **	−26.77 **		
P_3_ × P_6_	−4.23 **	−16.05 **	−7.14 **	−13.33 **	−8.70 **	−19.23 **		
P_4_ × P_5_	16.85 **	16.85 **	3.39 **	4.84 **	8.24 **	−7.07 **		
P_4_ × P_6_	8.94 **	8.06 **	−8.77 **	−16.13 **	−0.76 NS	−8.45 **		
P_5_ × P_6_	36.07 **	38.07 **	12.96 **	8.93 **	−14.09 **	−31.01 **		

DF—days to flowering, DM—days to maturity, MP—mid-parents, BP—better-parents and NS—Non-significant; *, ** Significant at 0.05 and 0.01 levels of probability, respectively.

**Table 6:** Soil properties at 0–15 cm soil depth.

Particle Size Distribution			Textural Class	Bulk Density (g/cm^3^)	pH	SOM (g/kg)	Total N (g/kg)	Exchangeable (Meq 100 g/soil)	Other Nutrients (mg/kg)			
Sand (%)	Silt (%)	Clay (%)							P	S	Zn	B
26	18	56	Clay loam	1.42	7.3	1.25	0.065	0.17	12	14	0.57	0.17

**Table 7:** Parents, pedigree, sources utilized in the investigation materials, and their exceptional highlights.

Sl. No.	Symbol	Parents/Cultivars	Pedigree	Sources	Special Features
1	P_1_	BMXK_1_-14004	Local cross	BARI, BD	High-yielding, medium seed, drought, and mungbean yellow mosaic virus (MYMV)-tolerant
2	P_2_	BARI Mung-1	Selection from NM92	BARI, BD	High-yielding, bold seed and MYMV-tolerant
3	P_3_	BINA Mung-8	MB149 with 400 Gy dose	BINA, BD	High-yielding, small seed, and MYMV-tolerant
4	P_4_	Sukumar	T-1 × K-441-11	IIPR, India	High-yielding, bold seed and MYMV-tolerant
5	P_5_	Pusa-7 (PS-7)	Selection from P-4092	IIPR, India	High-yielding, small seed, MYMV susceptible
6	P_6_	Sonali mung	Local	Local	Low-yielding, small seed and golden-colored, MYMV-tolerant

BARI, BD—Bangladesh Agricultural Research Institute, Bangladesh; BINA, BD—Bangladesh Institute of Nuclear Agriculture, Bangladesh; IIPR, India—Indian Institute of Pulses Research, India.

**Table 8:** Diallel crosses and their cross combinations.

Sl. No.	Crosses	Cross Combinations
1	P_1_ × P_2_	BMXK_1_-14004 × BARI Mung-1
2	P_1_ × P_3_	BMXK_1_-14004 × BINA Mung-8
3	P_1_ × P_4_	BMXK_1_-14004 × Sukumar
4	P_1_ × P_5_	BMXK_1_-14004 × Pusa-7 (PS-7)
5	P_1_ × P_6_	BMXK_1_-14004 × Sonali mung
6	P_2_ × P_3_	BARI Mung-1 × BINA Mung-8
7	P_2_ × P_4_	BARI Mung-1 × Sukumar
8	P_2_ × P_5_	BARI Mung-1 × Pusa-7 (PS-7)
9	P_2_ × P_6_	BARI Mung-1 × Sonali mung
10	P_3_ × P_4_	BINA Mung-8 × Sukumar
11	P_3_ × P_5_	BINA Mung-8 × Pusa-7 (PS-7)
12	P_3_ × P_6_	BINA Mung-8 × Sonali mung
13	P_4_ × P_5_	Sukumar × Pusa-7 (PS-7)
14	P_4_ × P_6_	Sukumar × Sonali mung
15	P_5_ × P_6_	Pusa-7 (PS-7) × Sonali mung

## Data Availability

Data recorded in the current study are available in all tables and figures of the manuscript.
